# Workload for radiologists during on-call hours: dramatic increase in the past 15 years

**DOI:** 10.1186/s13244-020-00925-z

**Published:** 2020-11-23

**Authors:** R. J. M. Bruls, R. M. Kwee

**Affiliations:** Department of Radiology, Zuyderland Medical Center, Henri Dunantstraat 5, 6419 PC Heerlen, The Netherlands

**Keywords:** Workload, After-hours care, Emergencies, Radiology

## Abstract

**Background:**

The objective of this study is to investigate the workload for radiologists during on-call hours and to quantify the 15-year trend in a large general hospital in Western Europe.

**Methods:**

Data regarding the number of X-ray, ultrasound and computed tomography (CT) studies during on-call hours (weekdays between 6.00 p.m. and 7.00 a.m., weekends, and national holidays) between 2006 and 2020 were extracted from the picture archiving and communication system. All studies were converted into relative value units (RVUs) to estimate the on-call workload. The Mann–Kendall test was performed to assess the temporal trend.

**Results:**

The total RVUs during on-call hours showed a significant increase between 2006 and 2020 (Kendall's tau-*b* = 0.657, *p* = 0.001). The overall workload in terms of RVUs during on-call hours has quadrupled. The number of X-ray studies significantly decreased (Kendall's tau-*b* = − 0.433, *p* = 0.026), whereas the number of CT studies significantly increased (Kendall's tau-*b* = 0.875, *p* < 0.001) between 2006 and 2020. CT studies which increased by more than 500% between 2006 and 2020 are CT for head trauma, brain CTA, brain CTV, chest CT (for suspected pulmonary embolism), spinal CT, neck CT, pelvic CT, and CT for suspected aortic dissection. The number of ultrasound studies did not change significantly (Kendall's tau-*b* = 0.202, *p* = 0.298).

**Conclusions:**

The workload for radiologists during on-call hours increased dramatically in the past 15 years. The growing amount of CT studies is responsible for this increase. Radiologist and technician workforce should be matched to this ongoing increasing trend to avoid potential burn-out and to maintain quality and safety of radiological care.

## Key points

- The number of CT studies during on-call hours increased significantly in the past 15 years.
- The overall on-call workload, as expressed in relative value units, increased fourfold between 2006 and 2020.
- Solutions should be sought to maintain acceptable workload levels.

## Background

A considerable part of emergency radiology studies is performed during on-call hours. It is the radiologist’s task to accurately interpret these studies and to communicate the findings in a timely manner with the referring physician. Higher volumes and complexity of cases put increasing pressure on radiologists to read more studies in a shorter period [1]. This results in longer working hours and reading fatigue, all of which contribute to diagnostic error [1–3]. Diagnostic errors are a major source of patient harm and result in death more often than any other medical error [1]. Emergency radiology studies during on-call hours may be particularly prone to diagnostic error due to relative staff shortage and absence of subspecialty trained attending radiologists. In addition, long shifts and high workloads are considered stressful, have negative health effects and can lead to burnout among radiologists [3, 4] and also among radiology technicians [5, 6]. Previous studies published in the early 2000s have demonstrated a 22% increase of radiological examinations during on-call hours over a four-year period in the USA [7] and an 85% increase over an eight-year period in the UK [8]. To our knowledge, there are no recently published studies that have quantified to which extent the workload for radiologists has increased and which type of studies contribute most to the workload during on-call hours. A current overview of the workload for radiologists during on-call hours is required to establish reasonable and safe benchmarks. This is relevant to both individual radiology departments to adjust staff accordingly and to national authorities who are responsible for the number of radiologists being trained. Therefore, the aim of our study was to investigate the current workload for radiologists during on-call hours and to quantify the 15-year trend in a large general hospital in Western Europe.

## Methods

This retrospective study was approved by the institutional review board of our hospital (Zuyderland Medical Center, Heerlen/Sittard-Geleen, The Netherlands) (No. Z2020061) and patients’ consents were waived.

### Data extraction

A search was conducted in the Picture Archiving and Communication System of Zuyderland Medical Center, which is one of the largest general teaching hospitals in The Netherlands with a service area which encompasses an estimated population of 420,000 [9]. All X-ray, ultrasound (US) and CT studies that were performed during on-call hours in the entire month of January of the years 2006 through 2020 were included. Our on-call hours were on weekdays between 6.00 p.m. and 7.00 a.m., in weekends, and on national holidays. Magnetic resonance imaging (MRI) studies were excluded since the far majority of these studies were performed in an outpatient setting in the evening or in the weekend. The total number of radiological studies per imaging modality (XR, US and CT) was extracted. For CT, the number of procedures per body part (head, paranasal sinuses, neck, spine, chest, abdomen, extremities, pelvis, and extracranial vasculature) and the number of interventional procedures (i.e., CT-guided percutaneous drainage) were extracted. Separate data extraction was performed for studies that were performed between 0.00 and 7.00 a.m., because of the possible negative implications on the circadian rhythm and sleep disruption [10, 11] for radiologists and radiology technicians.

### Conversion of number of studies to relative value units (RVUs)

All X-ray, US and CT studies were converted to RVUs, which are a composite measure of the time, complexity, and resources associated with a study or procedure. RVUs were adopted from the 2020 RVU list of the healthcare authority of The Netherlands [12].

### Statistical analysis

The Mann–Kendall test was performed to assess the temporal trend of the number of radiological studies along with the amount of RVUs during on-call hours over a 15-year period (2006–2020). Sub analysis was performed to investigate which procedures significantly increased over the past 15 years. Statistical analyses were performed using IBM SPSS version 26.0 (IBM Corporation). *P* values of less than 0.05 were regarded as statistically significant.

## Results

**Fig. 1:**
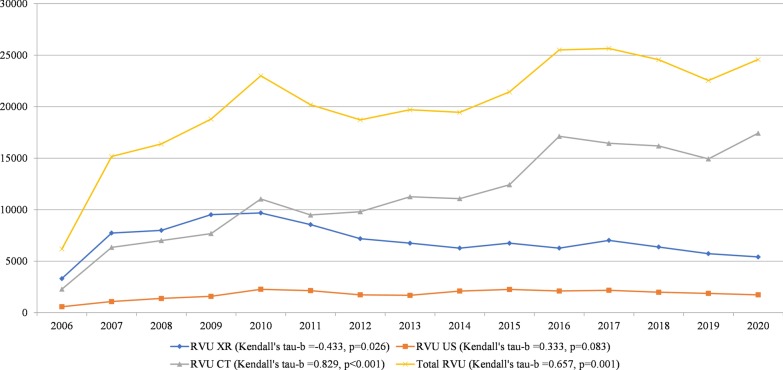
Relative value units of on-call studies per modality. Temporal trend of the relative value units of on-call studies per modality (XR, US, and CT) and of all modalities together, for the entire month of January of the years 2006 through 2020

The total RVUs during on-call hours (RVUs of all modalities added up) showed a significant increase between 2006 and 2020 (Kendall's tau-*b* = 0.657, *p* = 0.001 (Table 1 and Fig. 1). The overall workload during on-call hours increased by 297% (from 6187 RVU in January 2006 to 24,584 RVU in January 2020).

**Table 1:** Relative value units

	RVU XR	RVU US	RVU CT	Total RVU
2006	3315	586	2286	6187
2007	7740	1083	6350	15,172
2008	7998	1384	7005	16,387
2009	9519	1593	7685	18,796
2010	9687	2275	11,039	23,000
2011	8559	2143	9486	20,188
2012	7188	1735	9804	18,727
2013	6753	1687	11,262	19,702
2014	6273	2102	11,079	19,454
2015	6753	2257	12,426	21,436
2016	6273	2108	17,129	25,510
2017	7026	2177	16,451	25,653
2018	6381	1988	16,191	24,560
2019	5733	1878	14,934	22,545
2020	5415	1737	17,432	24,584

Relative value units of on-call studies for the entire month of January of the years 2006 through 2020

*RVU* relative value units, *US* ultrasound, *CT* computed tomography

**Fig. 2:**
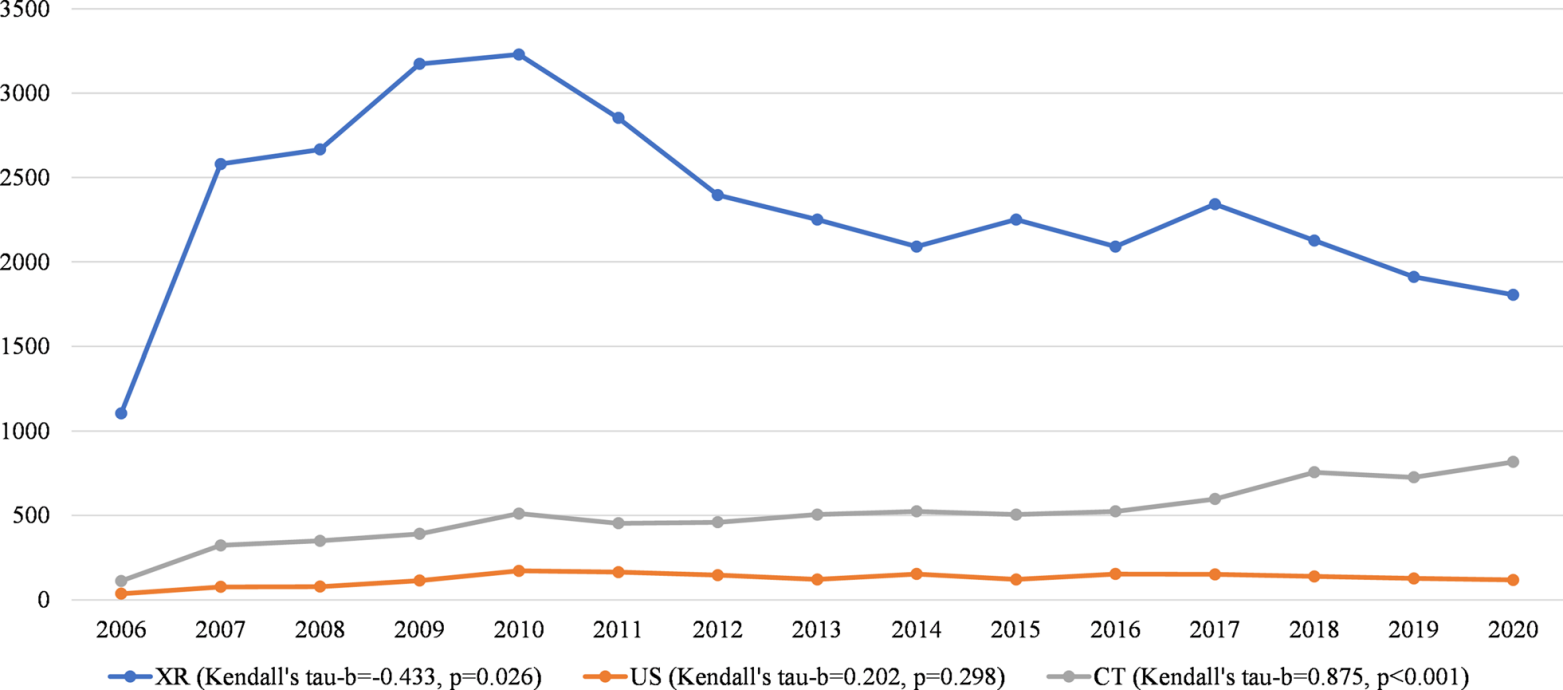
Number of on-call studies per modality. Temporal trend of the number of on-call studies per modality (XR, US, and CT) for the entire month of January of the years 2006 through 2020

**Fig. 3:**
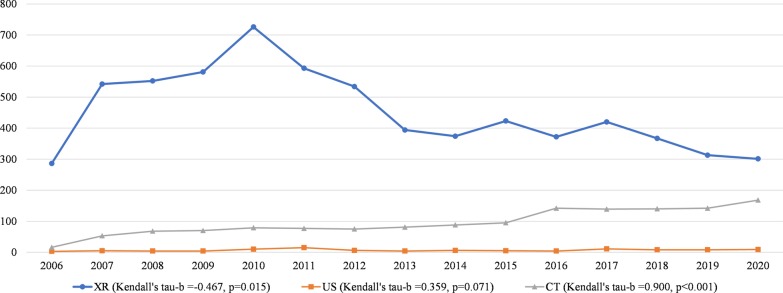
Number of on-call studies per modality between 0.00 and 7.00 am. Temporal trend of the number of on-call studies per modality (XR, US, and CT) between 0.00 and 7.00 am, for the entire month of January of the years 2006 through 2020

**Fig. 4:**
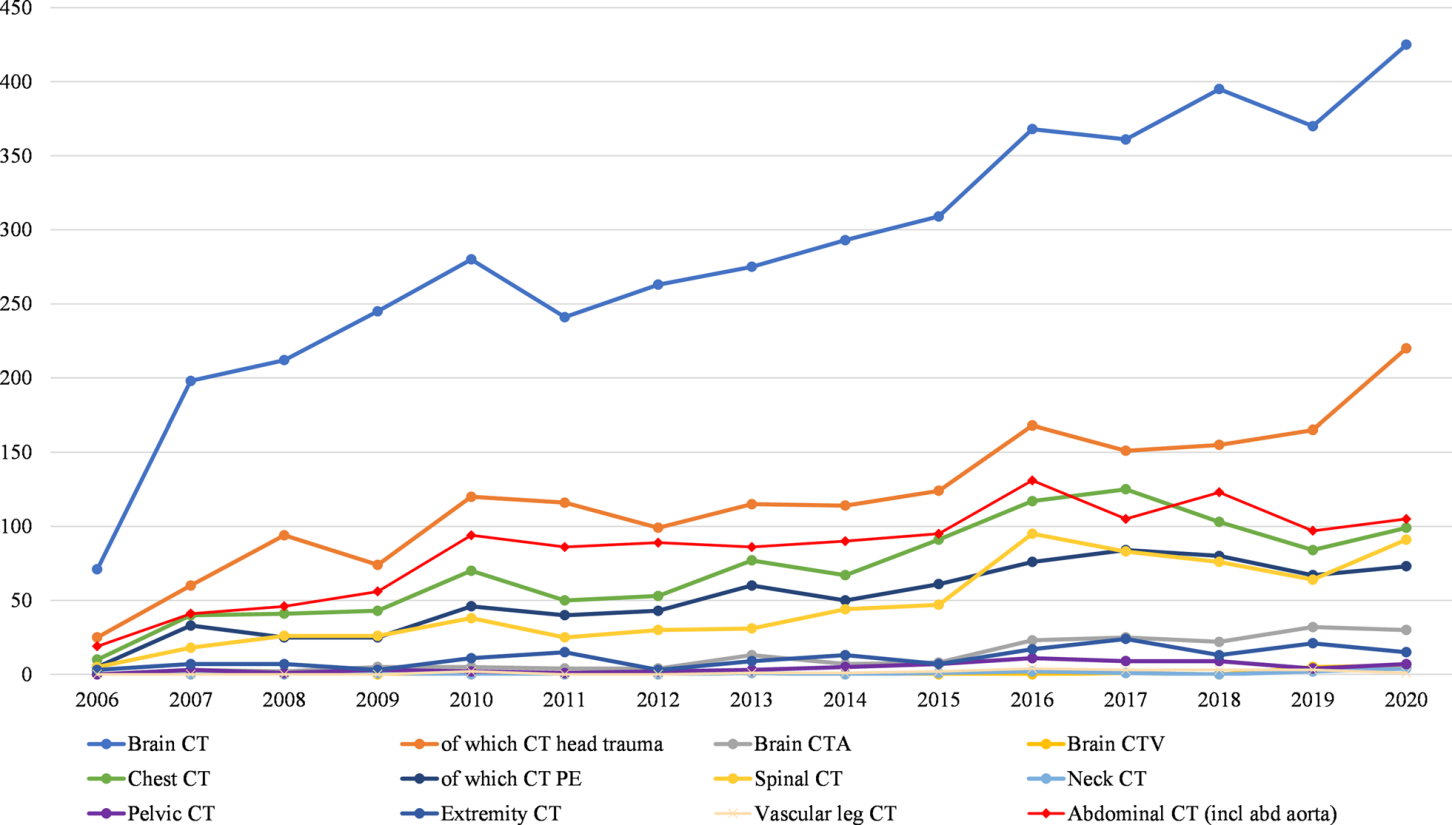
Number of CT procedures during on-call hours. Temporal trend of CT procedures during on-call hours, for the entire month of January of the years 2006 through 2020. Only CT procedures which significantly increased are displayed in the graph

Between 2006 and 2020, the number of X-ray studies during on-call hours significantly decreased (Kendall's tau-*b* = − 0.433, *p* = 0.026), whereas the number of US studies during on-call hours did not significantly change (Kendall's tau-*b* = 0.202, *p* = 0.298), and the number of CT studies significantly increased (Kendall's tau-*b* = 0.875, *p* < 0.001) (Table 2 and Fig. 2). Sub analysis of the studies that were performed between 0.00 and 7.00 a.m. showed similar trends: the number of X-ray studies significantly decreased (Kendall's tau-*b* = − 0.467, *p* = 0.015), whereas the number of US studies did not significantly change (Kendall's tau-*b* = − 0.359, *p* = 0.071), and the number of CT studies significantly increased (Kendall's tau-*b* = 0.900, *p* < 0.001) (Fig. 3). CT studies which increased by more than 500% between 2006 and 2020 were CT for head trauma, brain CTA, brain CTV, chest CT (for suspected pulmonary embolism), spinal CT, neck CT, pelvic CT and CT for suspected aortic dissection. Chest CT for suspected pulmonary embolism even increased by 1360% and spinal CT increased by 1720%. In 2015, brain CTA became the standard of care to assess intracranial large vessel occlusion in patients with acute ischemic stroke. This is represented by a 2.9 fold increase in brain CTA brain between January 2015 and January 2016 (from *n* = 8 to *n* = 23 per month) (Fig. 4). Subanalysis of all X-ray, ultrasound and CT studies that were performed during regular hours in the month January of 2006 through 2020 again showed similar results. The number of X-ray studies in January significantly decreased between 2006 and 2020 (Kendall’s tau-*b* = − 0.676, *p* < 0.001). The number of CT studies significantly increased (Kendall’s tau-*b* = 0.905, *p* < 0.001) and the number of ultrasound studies did not show a significant change (Kendall’s tau-*b* = − 0.238, *p* = 0.216).

**Table 2:** Number of on-call studies during the last 15 years

	XR	US	CT	Total
2006	1105	36	112	1253
2007	2580	77	323	2980
2008	2666	78	350	3094
2009	3173	114	391	3678
2010	3229	172	511	3912
2011	2853	164	454	3471
2012	2396	146	460	3002
2013	2251	121	505	2877
2014	2091	153	524	2768
2015	2251	121	505	2877
2016	2091	153	524	2768
2017	2342	151	597	3090
2018	2127	139	756	3022
2019	1911	127	726	2764
2020	1805	118	817	2740

Number of on-call studies for the entire month of January of the years 2006 through 2020

*US* ultrasound, *CT* computed tomography

## Discussion

Our study shows that the overall on-call workload for radiologists in terms of RVUs has quadrupled in the past 15 years. There has been an increasing trend in the number of CT studies that are performed during on-call hours between January 2006 and January 2020. During the same time period, the number of X-rays studies has significantly decreased, whereas the number of US studies has not changed significantly. CT studies are considered more time-consuming and more complex than X-ray studies, which explains the dramatic increase in overall workload. The largest increase has been seen in chest CT for pulmonary embolism and spinal CT.

Although the on-call workload has significantly increased, the number of radiology staff during on-call hours in our hospital has not increased. As a consequence, the individual workload has increased in the past 15 years. The workload during on-call hours is unlikely to stabilize let alone decrease in the near future, which is worrisome. Radiologists experience high rates of burnout and this trend has only been increasing [4, 13, 14]. This also applies to radiology technicians. High levels of burnout amongst radiologists can in turn be detrimental to quality and patient safety [4, 13, 14]. Solutions should be sought to maintain acceptable workload levels. The results of our study may be considered by national authorities who are responsible for the number of radiologists who are being trained. If the relative workload for radiologists or the outflow of radiologists becomes larger, more radiologists need to be trained to maintain a high quality of patient care. On a department level, possible solutions to lower the workload could be the deployment of physician assistants (who may take over some standard tasks of the radiologist), outsourcing part of the work through teleradiology [15], or the use of artificial intelligence [16, 17]. There are currently no uniformly defined maximum workload levels for radiologists during on-call hours. However, individual radiology departments could assess the current on-call workload and use the on-call workload in previous years as reference. By continuously monitoring the on-call workload, radiology departments can implement workload-reducing strategies in a timely manner. Medical imaging overutilization is a well-known problem which may have contributed to the increasing on-call workload [18–21]. Radiologists and radiology departments can play an active role to decrease medical imaging overutilisation by providing education and feedback to referring physicians [22, 23].

Our study shows that the workload during on-call hours has dramatically increased in the past 15 years. Possible explanations for this increase include easier access to CT, the introduction of new guidelines that involve imaging (such as brain CTA for detection of large vessel occlusion in patients with acute ischemic stroke) [24], patient pressures and the practise of defensive medicine [21, 25] which leads to imaging overutilisation. A study that was performed by Caroll in the United States and published in 2003 showed increasing numbers of on-call X-ray, US, CT and MRI studies from 1998 to 2002 with a total increase in the number of radiological studies during on-call hours of 22% in three years [7]. A study from 2006 that was performed by Herron et al. in the United Kingdom reported an 85% increase in the number of radiological studies during on-call hours in seven years [8]. This corresponds to an increase of 6.9% per year in the first study and 9.2% per year in the second study. Our data show an average increase of 5.7% per year of X-ray, US and CT studies combined. A limitation of the relatively old studies of Caroll et al. [7] and Herron et al. [8] is that they did not evaluate the overall workload in terms of RVUs. Our study also assessed the RVUs, which may be a better estimation of the increase of the overall workload.

Our study has some limitations. First, the current study was performed in one of the largest general hospitals (our hospital’s catchment area is a population of about 420,000 people) of a country which is considered to have one of the best health care systems in Europe [26]. Therefore, it is not sure whether our results can be generalized to smaller hospitals and hospitals in other countries. Second, we did not assess whether the amount of diagnostic errors simultaneously increased with the increasing workload. However, accurately measuring errors is difficult and subjective without a standard of reference [27]. In addition, it is well known that increasing workload results in longer working hours and reading fatigue, all of which contribute to diagnostic error [1–3, 28, 29]. Besides fatigue, circadian misalignment may also contribute to diminished diagnostic performance of radiologists [30]. Third, we only assessed the number of imaging studies but not the frequency, duration, and number of consultations with referring physicians (either by phone or direct contact), which also contribute to the workload for radiologists.

In conclusion, this study shows a dramatic increase in the workload for radiologists during on-call hours in the past 15 years. The growing amount of CT studies is responsible for this increase. Radiologist and technician workforce should be matched to this ongoing increasing trend to avoid potential burn-out and to maintain quality and safety of radiological care.

## Data Availability

The datasets used and/or analyzed during the current study are available from the corresponding author on reasonable request.

## Abbreviations

CT: Computed tomography
MRI: Magnetic resonance imaging
RVU: Relative value unit
US: Ultrasound
XR: X-ray
